# The impact of SASA!, a community mobilization intervention, on reported HIV-related risk behaviours and relationship dynamics in Kampala, Uganda

**DOI:** 10.7448/IAS.17.1.19232

**Published:** 2014-11-05

**Authors:** Nambusi Kyegombe, Tanya Abramsky, Karen M Devries, Elizabeth Starmann, Lori Michau, Janet Nakuti, Tina Musuya, Lori Heise, Charlotte Watts

**Affiliations:** 1 Department of Global Health and Development, London School of Hygiene and Tropical Medicine, London, UK; 2 Raising Voices, Kampala, Uganda; 3 Centre for Domestic Violence Prevention, Kampala, Uganda

**Keywords:** HIV-related risk behaviours, relationship dynamics, intimate partner violence, SASA!, community mobilization, Uganda

## Abstract

**Introduction:**

Intimate partner violence (IPV) violates women's human rights, and it is a serious public health concern associated with increased HIV risk. SASA!, a phased community mobilization intervention, engages communities to prevent IPV and promote gender equity. The SASA! study assessed the community-level impact of SASA! on reported HIV-related risk behaviours and relationship dynamics.

**Methods:**

Data were collected as part of a cluster randomized controlled trial conducted between 2007 and 2012 in eight communities in Kampala. An adjusted cluster-level intention to treat analysis, compares secondary outcomes in intervention and control communities at follow-up. The qualitative evaluation explored participants’ subjective experience of SASA!. A total of 82 in-depth interviews were audio recorded at follow-up, transcribed verbatim and analyzed using thematic analysis.

**Results:**

Men in intervention communities were significantly more likely than controls to report a broad range of HIV-protective behaviours, including higher levels of condom use (aRR 2.03, 95% CI 1.22–3.39), HIV testing (aRR 1.50, 95% CI 1.13–2.00) and fewer concurrent partners (aRR 0.60, 95% CI 0.37–0.97). They were also more likely to report increased joint decision-making (aRR 1.92, 95% CI 1.27–2.91), greater male participation in household tasks (aRR 1.48, 95% CI 1.09–2.01), more open communication and greater appreciation of their partner's work inside (aRR 1.31, 95% CI 1.04–1.66) and outside (aRR 1.49, 95% CI 1.08–2.06) the home. For women, all outcomes were in the hypothesized direction, but effect sizes were smaller. Only some achieved statistical significance. Women in intervention communities were significantly more likely to report being able to refuse sex with their partners (aRR 1.16, 95% CI 1.00–1.35), joint decision-making (aRR 1.37, 95% CI 1.06–1.78) and more open communication on a number of indicators. Qualitative interviews suggest that shifts operated through broader improvements in relationships, including increased trust and cooperation, participants’ greater awareness of the connections between HIV and IPV and their resultant desire to improve their relationships. Barriers to change include partial uptake of SASA!, partner resistance, fear and entrenched previous beliefs.

**Conclusions:**

SASA! impacted positively on reported HIV-related risk behaviours and relationship dynamics at a community level, especially among men. Social change programmes focusing on IPV and gender equity could play an important role in HIV prevention efforts.

## Introduction

Recent global estimates indicate that nearly one in three women experience physical or sexual violence from an intimate partner in their lifetime [1]. As well as being a violation of women's human rights, violence against women is a serious public health concern [2–5]. Through both direct [6] and indirect mechanisms [6–8], intimate partner violence (IPV) can increase women's vulnerability to HIV infection [7], with recent population-based cohort studies in Uganda and South Africa demonstrating an association with incident HIV infection [7,9]. Gender inequality reduces women's ability to negotiate sex or insist on condom use, and thus their ability to protect themselves from infection. In sub-Saharan Africa, women constitute 58% of those living with HIV [10]. Women diagnosed with HIV may also be at increased risk of violence, which, together with the fear of violence, may prevent women from testing, disclosing their status or pursuing treatment [7,10–13].

**Figure 1:**
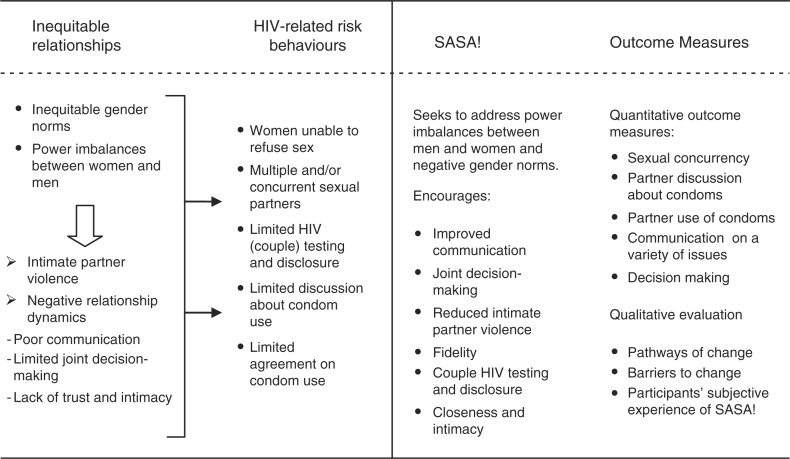
SASA!'s intended impact on HIV-related risk behaviours and relationship dynamics.

There is growing evidence that participatory, gender transformative violence prevention programmes can both impact levels of IPV and reduce HIV-related risk behaviours [14–17]. Currently, this evidence comes primarily from research assessing impact on direct intervention recipients. Shifts in HIV-risk behaviours may result directly from reduced violence (or the threat of it) within intimate partnerships, for example, if the incidence of coerced sex declines [18] or women no longer feel afraid to request condom use or discuss HIV testing [19]. They may also follow from the broader changes to relationship dynamics. More equitable relationships manifested through, for example, improved communication and joint decision-making [20] may provide environments in which violence is less likely, but also where discussion about HIV and protective behaviours is possible and desirable [18,19]. Multiple partnerships may also be less sought out [17]. Figure 1 illustrates these pathways in more detail.

**Figure 2:**
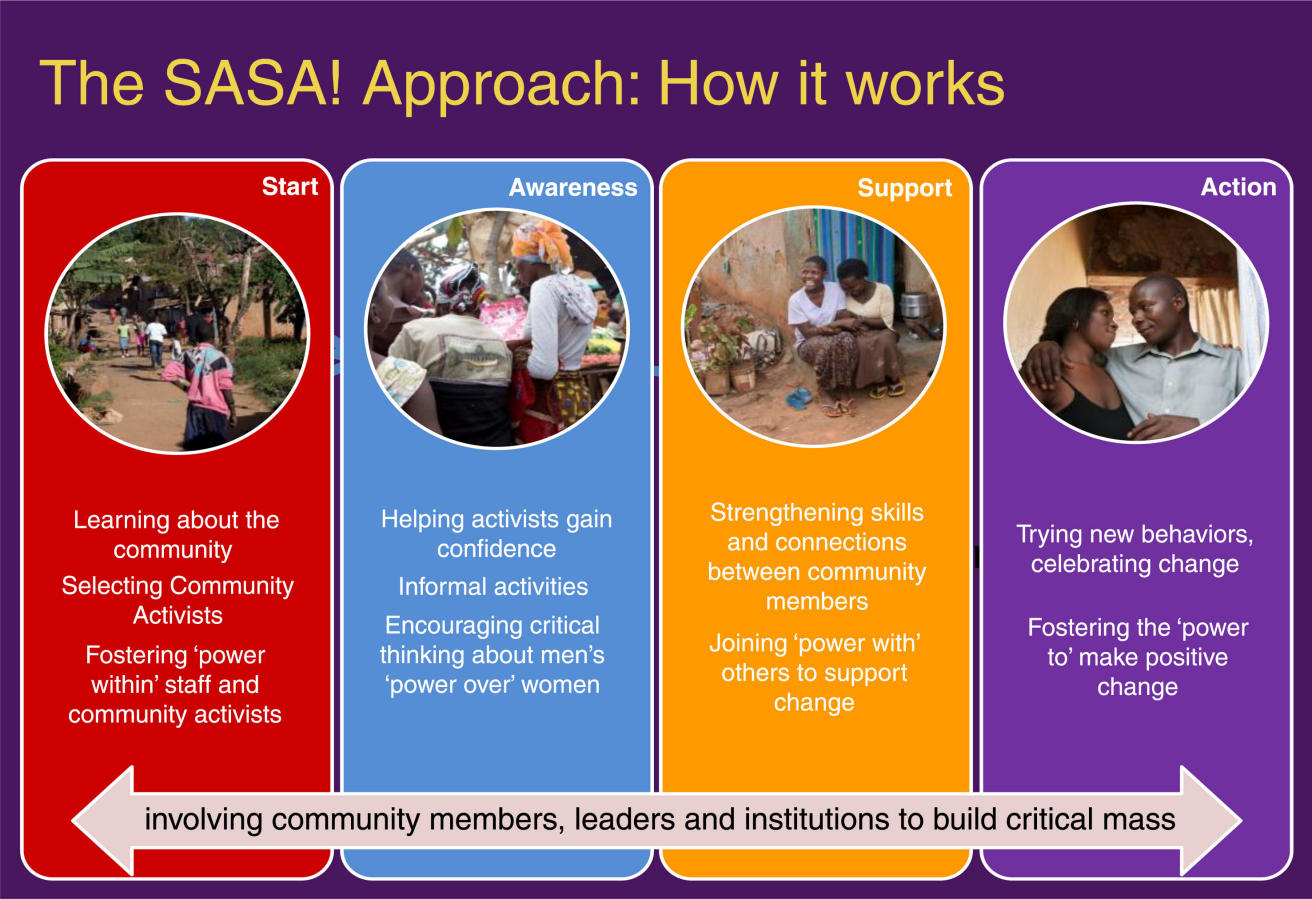
The four phases of SASA!.

The primary trial analysis showed the intervention (summarised in Box 1) to be associated with lower past year experience of physical and sexual IPV among women and lower levels of sexual concurrency among men [22]. This paper presents findings on SASA!'s impact on secondary outcomes relating to HIV-related risk behaviours and several indicators of relationship dynamics. The qualitative findings explore pathways and barriers to change and participants’ subjective experiences of, and views on, SASA! programming.

## Methods

### Study design

As described in detail elsewhere [23], the SASA! study is a pair-matched cluster randomized controlled trial with baseline and end line cross-sectional surveys, a nested qualitative study, on-going operational research and an economic evaluation. The research was conducted between 2007 and 2012 in two administrative divisions of Kampala, Uganda. Kampala has a high prevalence of poverty, HIV/AIDS and IPV, with 52.3% of women aged 15–49 estimated to have lifetime experience of physical or sexual partner violence, and 9.5% to be living with HIV [24,25]. Patriarchy is a dominant aspect of the socio-cultural environment.

**Box 1. What is SASA!?**

SASA! is a phased community mobilization intervention that seeks to prevent IPV and reduce HIV-related risk behaviours at the community level. This is achieved using a community mobilization intervention that, through social diffusion, focuses upon shifting harmful social norms, and on addressing the power imbalances between women and men that perpetuate violence, HIV-related risk and inequitable relationships [21]. SASA! was designed by Raising Voices and is implemented in Kampala by the Centre for Domestic Violence Prevention (CEDOVIP), both of which are Uganda-based NGOs. Appendix 1 provides a detailed description of the intervention and the implementing organizations. In brief, SASA! entails selecting and supporting community members to actively discuss and engage on issues of gender inequality, violence and HIV. Community members include “community activists” (a selection of ordinary community members that receive on-going support and training to implement the intervention); professionals including healthcare workers and police; and local cultural and government leaders. Activists work on a voluntary, unpaid basis and are supported by CEDOVIP through regular training and mentoring to conduct a variety of activities to engage women and men, groups and institutions within the community. SASA!'s programming aims to be aspirational, with a benefits-based approach. Figure 2 outlines the four phases of SASA!

### Survey sampling

**Figure 3:**
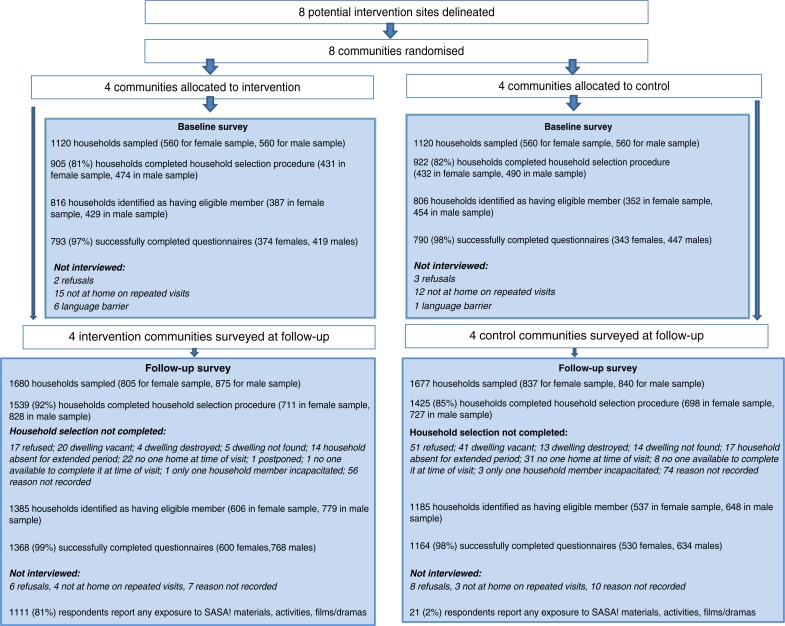
Consort diagram of the SASA! Study.

The study area comprised eight sites, four intervention and four controls (Figure 3). Two cross-sectional surveys of community members were undertaken, one at baseline, one four years later. Within each randomly selected household, one eligible member was randomly selected for interview. A total of 1583 respondents were interviewed at baseline and 2532 at follow-up (due to a larger budget allowing more households to be sampled). The survey was designed to assess the community-level impact of SASA! on a number of primary and secondary outcomes related to IPV, HIV-related risk behaviours and relationship dynamics.

### Measures

Outcomes were constructed as binary measures (Appendix 2). Questions on sexual IPV were the same as those used in the WHO Multi-Country Study on Women's Health and Domestic Violence [26] and similar to those in the Uganda Demographic and Health Survey [24]. All items were cognitively pretested and refined based on successive rounds of testing.

### Analysis

Quantitative analyses were conducted using STATA 12.0 [27]. Analyses were carried out separately for male and female respondents, reflecting *a priori* assumptions that both outcome prevalence and intervention effects would differ between men and women. A cluster-level intention to treat (ITT) analysis was used to compare outcomes in intervention and control communities at follow-up. The crude intervention effect was estimated using the geometric mean prevalence ratio. Statistical weighting was used to account for differences in the denominators between sites. Adjusted risk ratios were calculated from ratios of observed to expected outcomes in intervention and control sites – site-level expected prevalence figures predicted by fitting a logistic regression model to individual-level data, with the outcomes as the dependent variables, and age, marital status and baseline enumeration area-level prevalence of the outcome measure of interest as the independent variables.

A per-protocol analysis was also performed using a similar approach to the ITT analysis, but including only those with at least a moderate level of exposure to SASA! in the intervention site-level summaries, and controls matched on propensity for intervention exposure in the control site-level summaries [23].

### Qualitative study

The qualitative evaluation explored participants’ subjective experience of the intervention and its impact on intimate relationship dynamics, including HIV-related risk behaviours. A total of 82 in-depth interviews with community members, (20 women and 20 men) community activists (10 women and 10 men) and community leaders (six women and six men) were conducted at follow-up using a semi-structured tool. Community members were purposively sampled from among survey respondents to reflect those who noted reduced violence in the past 12 months as compared to the period before. Community activists and local leaders were purposively sampled upon the advice of CEDOVIP and Raising Voices staff to reflect individuals with varying experiences of implementing SASA!. Qualitative interviews were audio recorded, transcribed verbatim and analyzed using thematic analysis assisted by NVIVO 10 [28].

### Study ethics

The study adhered to guidelines provided by the WHO for safe and ethical collection of data on violence against women [29]. All respondents provided written informed consent and were interviewed in a safe and private place of their choice. For reasons of participant safety and logistics, in each enumeration area, only men or women were interviewed. Only one respondent per sampled household was interviewed. Ethical approval was obtained from the London School of Hygiene and Tropical Medicine, Makerere University and Uganda National Council of Science and Technology. Study registration at ClinicalTrials.gov(NCT00790959).

## Results

Characteristics of survey respondents are summarized in Table 1. Data indicate a high level of comparability between intervention and control communities with respect to socio-demographic characteristics at both baseline and follow-up. Tables 2 and 3 also demonstrate baseline comparability between intervention and control communities for the outcome indicators.

**Table 1:** Characteristics of study respondents

	Baseline				Follow-up			
		
	Intervention		Control		Intervention		Control	
				
	Men	Women	Men	Women	Men	Women	Men	Women
Household-level	*N*=419	*N*=374	*N*=447	*N*=343	*N*=768	*N*=599	*N*=634	*N*=529
Household has electricity	328 (78%)	259 (69%)	367 (82%)	264 (77%)	675 (88%)	503 (84%)	544 (86%)	445 (84%)
Main drinking water source – public tap	267 (64%)	228 (61%)	324 (72%)	212 (62%)	559 (73%)	391 (65%)	452 (71%)	336 (64%)
Toilet facility – traditional pit toilet/latrine	281 (67%)	225 (60%)	268 (60%)	203 (59%)	415 (54%)	389 (65%)	351 (55%)	302 (57%)
House is rented	279 (67%)	231 (62%)	310 (69%)	246 (72%)	622 (81%)	448 (75%)	484 (76%)	379 (72%)
House is in gated compound	–	–	–	–	66 (9%)	78 (13%)	81 (13%)	118 (22%)
Individual-level								
Age (years)	27.1 (6.8)	28.4 (7.7)	27.6 (7.0)	28.2 (7.7)	28.6 (7.8)	28.4 (7.4)	29.9 (8.2)	29.1 (8.2)
Lived in same zone since before aged 12 (baseline)/for longer than 3 years (follow-up)	84 (20%)	44 (12%)	94 (21%)	45 (13%)	610 (79%)	353 (59%)	466 (74%)	313 (59%)
Muganda (tribe)	304 (73%)	263 (71%)	307 (69%)	202 (59%)	514 (67%)	373 (62%)	369 (58%)	315 (60%)
Main religions								
Catholic	164 (39%)	119 (32%)	177 (40%)	108 (31%)	284 (37%)	209 (35%)	237 (37%)	165 (31%)
Muslim	103 (25%)	90 (24%)	114 (26%)	93 (27%)	184 (24%)	140 (23%)	158 (25%)	123 (23%)
Protestant	79 (19%)	104 (28%)	107 (24%)	80 (23%)	207 (27%)	141 (24%)	171 (27%)	133 (25%)
Born again	52 (12%)	50 (13%)	34 (8%)	49 (14%)	75(10%)	97 (16%)	51 (8%)	97 (18%)
Above primary education	275 (66%)	157 (42%)	321 (72%)	140 (41%)	556 (72%)	394 (66%)	457 (72%)	343 (65%)
Able to read	399 (95%)	345 (92%)	429 (96%)	313 (92%)	735 (96%)	535 (89%)	581 (92%)	480 (91%)
Does not earn money	87 (21%)	180 (48%)	94 (21%)	166 (48%)	108 (14%)	219 (37%)	63 (10%)	177 (33%)
Ever had a regular partner	326 (78%)	350 (94%)	352 (79%)	316 (92%)	584 (76%)	558 (93%)	481 (76%)	487 (92%)
Including casual					689 (90%)	574 (96%)	573 (90%)	497 (94%)
Had a regular partner in the past 12 months	313 (75%)	305 (82%)	335 (75%)	274 (80%)	545 (71%)	486 (81%)	435 (69%)	401 (76%)
Including casual					624 (81%)	504 (84%)	525 (83%)	427 (81%)
Currently married/cohabiting	165 (39%)	228 (61%)	191 (43%)	205 (60%)	407 (53%)	377 (63%)	314 (50%)	286 (54%)
In polygamous marriage (among those married)	37/165 (22%)	49/201 (24%)	45/191 (24%)	57/187 (30%)	36/407 (9%)	53/316 (17%)	38/314 (12%)	57/246 (23%)
No children	237 (57%)	83 (22%)	223 (50%)	83 (24%)	351 (46%)	136 (23%)	267 (42%)	121 (23%)

**Table 2:** Estimates of effect on HIV-related outcome indicators among women who had a regular partner in the past year,^a^ comparing prevalence of outcome in intervention versus control communities

	Baseline^b^		Follow-up		Unadjusted RR^a^ (95% CI)	Adjusted RR^c^ (95% CI)
	
	Intervention	Control	Intervention	Control		
HIV risk behaviours						
Past year experience of sexual IPV	33/254 (13%)	20/216 (9%)	60/433 (14%)	66/343 (19%)	0.81 (0.32–2.05)	0.81 (0.31–2.10)
Feels able to refuse sex with partner	244/254 (96%)	203/216 (94%)	403/428 (94%)	277/341 (81%)	**1.16 (1.00–1.35)**	**1.16 (1.00–1.35)**
Either respondent or partner initiated discussion about condom use	139/247 (56%)	110/214 (51%)	242/429 (56%)	178/341 (52%)	1.08 (0.91–1.27)	*1.11 (0.99–1.25)*
Used condom in past year	71/244 (29%)	76/214 (36%)	164/429 (38%)	112/341 (33%)	1.15 (0.79–1.69)	1.22 (0.90–1.66)
Used condom at last intercourse	40/246 (16%)	38/214 (18%)	82/429 (19%)	49/341 (14%)	1.37 (0.59–3.20)	1.58 (0.86–2.89)
Respondent had concurrent partner in past year	18/245 (7%)	8/215 (4%)	25/429 (6%)	20/341 (6%)	1.16 (0.33–4.09)	1.25 (0.37–4.22)
Discussed HIV testing with partner in past year	184/248 (74%)	169/214 (80%)	338/429 (79%)	251/341 (74%)	1.07 (0.98–1.17)	1.09 (0.94–1.27)
Respondent had HIV test in past year	133/254 (52%)	118/217 (54%)	344/432 (80%)	269/348 (77%)	1.01 (0.92–1.12)	1.02 (0.89–1.15)
Relationship dynamics						
Made important decisions jointly with partner all/most of the time	182/217 (84%)	162/189 (86%)	248/368 (67%)	133/275 (48%)	**1.41 (1.06–1.86)**	**1.37 (1.06–1.78)**
Male partner helps with housework	134/241 (56%)	137/215 (64%)	245/340 (72%)	143/242 (59%)	1.24 (0.89–1.74)	1.24 (0.90–1.71)
Male partner helps look after children	147/211 (70%)	125/166 (75%)	203/286 (71%)	133/209 (64%)	1.12 (0.78–1.62)	1.10 (0.78–1.54)
Shown appreciation many times for work partner does in the house			237/345 (69%)	135/247 (55%)	**1.28 (1.03–1.58)**	*1.22 (0.99–1.50)*
Shown appreciation many times for work partner does outside the house			301/350 (86%)	201/252 (80%)	1.08 (0.97–1.20)	1.06 (0.97–1.16)
Discussed number of children you would like to have			319/427 (75%)	237/340 (70%)	1.07 (0.91–1.26)	1.05 (0.90–1.22)
Openly asked what partner likes during sex			295/428 (69%)	167/341 (49%)	1.42 (0.90–2.24)	1.45 (0.93–2.25)
Openly told partner what you like during sex			325/428 (76%)	192/341 (56%)	1.36 (0.89–2.07)	1.37 (0.90–2.08)
Discussed things that happen to both you and partner during the day	211/254 (83%)	194/217 (89%)	358/429 (83%)	233/341 (68%)	1.23 (0.98–1.54)	1.21 (0.96–1.53)
Discussed your worries/feelings	218/254 (86%)	194/217 (89%)	386/429 (90%)	259/341 (76%)	1.19 (0.99–1.19)	*1.18 (0.99–1.42)*

a Risk ratios calculated at the cluster-level, both crude and adjusted ratios adjusting for community-pair, and weighted according to the number of observations per village.

b Question wording/item construction changed between baseline and follow-up to improve face validity – those baseline measures closest to the follow-up outcomes are presented here to assess underlying intervention/control community comparability, but baseline/follow-up comparisons are not possible.

c Adjusted risk ratios generated on the basis of expected number of events from a logistic regression model on individual data with independent variables including age and marital status.

Bold values is used to indicate results that were statistically significant.

**Table 3:** Estimates of effect on HIV-related outcome indicators among men who had a regular partner in the past year,^a^ comparing prevalence of outcome in intervention versus control communities

	Baseline^b^		Follow-up		Unadjusted RR^a^ (95% CI)	Adjusted RR^c^ (95% CI)
	
	Intervention	Control	Intervention	Control		
HIV risk behaviours						
Past year experience of sexual IPV					–	–
Feels able to refuse sex with partner					–	–
Either respondent or partner initiated discussion about condom use	163/273 (60%)	173/285 (61%)	421/508 (83%)	230/397 (58%)	**1.43 (1.25–1.63)**	**1.46 (1.17–1.82)**
Used condom in past year	147/272 (54%)	164/285 (58%)	361/507 (71%)	187/397 (47%)	**1.52 (1.04–2.20)**	1.54 (0.96–2.47)
Used condom at last intercourse	97/273 (36%)	101/285 (35%)	210/508 (41%)	87/397 (22%)	**1.91 (1.13–3.23)**	**2.03 (1.22–3.39)**
Respondent had concurrent partner in past year	109/270 (40%)	104/283 (37%)	139/508 (27%)	177/397 (45%)	0.60 (0.35–1.02)	**0.60 (0.37–0.97)**
Discussed HIV testing with partner in past year	161/274 (59%)	157/286 (55%)	433/508 (85%)	246/397 (62%)	**1.39 (1.10–1.76)**	**1.37 (1.09–1.73)**
Respondent had HIV test in past year	109/276 (39%)	114/289 (39%)	415/507 (82%)	217/404 (54%)	**1.54 (1.15–2.05)**	**1.50 (1.13–2.00)**
Relationship dynamics						
Made important decisions jointly with partner all/most of the time	176/197 (89%)	188/216 (87%)	351/407 (86%)	146/318 (46%)	**1.88 (1.25–2.83)**	**1.92 (1.27–2.91)**
Male partner helps with housework	156/267 (58%)	178/284 (63%)	362/376 (96%)	204/288 (71%)	**1.38 (1.05–1.83)**	**1.38 (1.03–1.85)**
Male partner helps look after children	116/147 (79%)	148/189 (78%)	312/332 (94%)	162/256 (63%)	**1.49 (1.08–2.07)**	**1.48 (1.09–2.01)**
Shown appreciation many times for work partner does in the house			352/375 (94%)	204/288 (71%)	**1.35 (1.06–1.72)**	**1.31 (1.04–1.66)**
Shown appreciation many times for work partner does outside the house			204/258 (79%)	116/215 (54%)	**1.54 (1.12–2.12)**	**1.49 (1.08–2.06)**
Discussed number of children you would like to have			448/506 (89%)	276/396 (70%)	**1.28 (1.04–1.57)**	**1.25 (1.05–1.50)**
Openly asked what partner likes during sex			457/508 (90%)	223/396 (56%)	**1.61 (1.23–2.09)**	**1.59 (1.23–2.05)**
Openly told partner what you like during sex			463/508 (91%)	247/397 (62%)	**1.48 (1.16–1.88)**	**1.46 (1.16–1.85)**
Discussed things that happen to both you and partner during the day	239/276 (87%)	252/289 (87%)	487/509 (96%)	285/397 (72%)	**1.34 (1.02–1.77)**	1.32 (1.00–1.74)
Discussed your worries/feelings	238/276 (86%)	257/289 (89%)	489/509 (96%)	298/397 (75%)	**1.29 (1.02–1.64)**	**1.27 (1.01–1.60)**

a Risk ratios calculated at the cluster-level, both crude and adjusted ratios adjusting for community-pair, and weighted according to the number of observations per village.

b Question wording/item construction changed between baseline and follow-up to improve face validity – those baseline measures closest to the follow-up outcomes are presented here to assess underlying intervention/control community comparability, but baseline/follow-up comparisons are not possible.

c Adjusted risk ratios generated on the basis of expected number of events from a logistic regression model on individual data with independent variables including age and marital status.

Bold values is used to indicate results that were statistically significant.

### Impacts on men

Among men, effect estimates in the hypothesized direction were observed for all HIV-risk behaviours and indicators of relationship dynamics, with results statistically significant at the 5% level for all but two outcomes. Positive impacts include increased discussion about, and use of, condoms within regular partnerships in the past year with men in intervention communities twice as likely to report condom use with their partner at last intercourse than men in control communities (aRR 2.03, 95% CI 1.22–3.39). Levels of discussion around HIV testing and reports of having been tested in the past year were also higher, with men in intervention communities being 50% more likely to have had a test, relative to control counterparts (aRR 1.50, 95% CI 1.13–2.00). Reported sexual concurrency was significantly lower among men in intervention communities (aRR 0.60, 95% CI 0.37–0.97).

Men in intervention communities reported higher levels of joint decision-making than their control counterparts (aRR 1.92, 95% CI 1.27–2.91); greater participation in activities traditionally in the domain of women, such as looking after children (aRR 1.48, 95% CI 1.09–2.01); and greater appreciation for work done by their wives inside (aRR 1.31, 95% CI 1.04–1.66) and outside the home (aRR 1.49, 95% CI 1.08–2.06). They also reported more open communication than men in control communities, including in relation to discussion around sex, with intervention men approximately 60% more likely to have asked their partner what they like during sex (aRR 1.59, 95% CI 1.23–2.05); discussions around things that have happened to both partners during the day (aRR 1.32, 95% CI 1.00–1.74); and discussions about worries and feelings (aRR 1.27, 95% CI 1.01–1.60).

### Impacts on women

While effect estimates among women were in the hypothesized direction for all indicators, effect sizes were smaller than those for men with several point estimates close to 1 (no effect). Only two of the adjusted risk ratios were statistically significant at the 5% level, though several more were of borderline significance. For the most part, the results suggest that SASA! had little impact on HIV-risk-related behaviours among women, with the notable exception of a woman's ability to refuse sex: women in SASA! communities reported feeling more able to refuse sex with their partner (aRR 1.16, 95% CI 1.00–1.35). They were also less likely to report past year experience of sexual IPV (aRR 0.81, 95% CI 0.31–2.10) and more likely to report condom use with their partner at last intercourse (aRR 1.58, 95% CI 0.86–2.89), though neither result was statistically significant.

Effect sizes tended to be larger for indicators of relationship dynamics than for HIV-related risk behaviours with women in intervention communities significantly more likely than their control counterparts to report making decisions jointly with their partner (aRR 1.37, 95% CI 1.06–1.78). Other indicators did not reach statistical significance.

Results from the per-protocol analysis were similar to those from the ITT analysis. More effect estimates were statistically significant when the analysis was confined to women reporting SASA! exposure (and their control counterparts). These included discussion around condom use; partner helping with the housework; asking what their partner liked during sex; and discussing worries and feelings.

### Pathways of change

Findings from the survey were also separately reflected in those of the qualitative evaluation. As elaborated in a paper that reports on the qualitative evaluation of SASA!, to varying degrees, participants described how through engagement in SASA!, their relationships benefited from more supportive gender roles, improved communication, increased levels of joint decision-making and awareness of non-violent ways to deal with anger and disagreement. Not all relationships experienced the same breadth and depth of change, however, with some forms of violence persisting in some relationships [30].

For some participants, particularly those who were actively engaged with SASA!, shifting relationship dynamics related to communication; decision-making and agreement; and more deliberate efforts to co-operate, influenced HIV-related risk behaviours. In a number of relationships, the nature of communication had previously been functional for the day-to-day running of their households. SASA! encouraged deeper and more meaningful communication including about women's right to refuse sex:

We talk about bedroom issues. In those days before SASA!, when my husband wanted sex it was a must, I had to give it to him, but now, if I don't feel like having sex I will just tell him and he will understand. (CF1 Female)

This also reflected improvements in relationship quality and intimacy which for a few women had a profound impact on their feelings for their partner.

When you tell a person that “I do not want to have sex” and he forces you, within you, you feel disgusted with this person and even hate him. But for a person whom you tell that today I don't want to have sex with you and he listens, you feel deep down within you that you have started to trust, love and respect this person, because just by doing that, he has respected you. (CF19, Female)

With improving communication, many women described how their ability to make decisions with their partners also improved. This often had implications on HIV-protective behaviours including condom use.

In order to have sex you should discuss it first and agree with each other. That means that even if I bring a condom he will agree and accept it because we agree with each other in bed. SASA! has done a big job. It brought about agreeing and negotiating in the bedroom, on sex, discussing when to have it and even talking about condoms. (CA30F Female)

Increased willingness to discuss and use condoms was also reported by some men.

Improvements in communication and decision-making also resulted in a number of couples being more willing to test for HIV and share their results. Amongst these couples, learning that neither of them was living with HIV offered an opportunity to “reset their relationship” and renew a commitment to be faithful:

During the session the facilitators encouraged us to test for HIV so after the session I agreed and we went to test. The results came out well and from then we agreed to be faithful to each other. (CM9 Male)

Shifts in relationship dynamics and HIV-related risk behaviours were not universal. Summarized in Table 4, barriers to change included individuals’ choice not to implement aspects of SASA! in their lives; partners’ resistance to change; fear, particularly with relation to HIV testing; and religious and personal beliefs that inhibited change.

**Table 4:** Barriers to change

	Barriers to change
Partial uptake of some aspects of SASA!	“I have another woman outside my home. She is the only one I have outside my home. From the time of watching the SASA! film I decided to leave all the others [women] and remain with only two. I did it out of my own free will for the good of my health and my home.” (CM18 Male)
Resistance from partner	“I was worried because I asked him to go for an HIV test but he refused, but I tested and think I am safe because I have been testing ever since and my results have been negative so I assume he is also safe.” (CF1 Female) “I would have wanted him to attend [SASA! activities] so that he can learn. I want them to teach him instead of teaching me, may be if another person spoke with him, he could learn, I have brought him a book from SASA! but he refused to read it.” (CF5 Female)
Fear	“We tell them it takes little time and then at times we offer to give them recommendations for easy access points and you still follow them up and ask if they eventually went for the test but their wives tell you that they are still hesitant.” (CA6M Male)
Religion and beliefs	“Now let me tell you for us Born Again [Christians] it is very hard for us to leave a marriage but the truth is that if it was not that, I would have left this marriage a long time ago. He does not beat me but there is a way that he behaves that hurts me badly.” (CF5 Female)
CODES	CF (community member female) CM (Community member male) CA*F (community activist or local leader female) CA*M (community activist or local leader male)

The findings of the qualitative evaluation also illustrate why SASA! was perceived as different, and for some participants, more effective at motivating a change in their HIV-related risk behaviours than other HIV prevention efforts to which they have been exposed (Table 5).

**Table 5:** Why is SASA! different and more effective than other HIV prevention efforts?

Thought-provoking delivery	“For me it's the dramas that are most interesting because whenever they stage them you are able to relate to what they are staging and in most cases one will be given a chance to relate what they have seen to their real lives [and you see that] you also need to change your behaviours.” (CM8 Male) “In the drama they stage a skit and you are able to reflect on how you are moving on with your life and in that process you are able to revise those things that you have been doing in your life that could put you at risk of infection.” (CM10 Male)
Connection with IPV	“SASA! prevents domestic violence. Most of the time we get HIV from domestic violence. I can leave home, when my husband [has] annoyed me and I say, let me go to my extra marital partner, so that he can calm me down [but] I don't know his [partner's] movements so if violence is prevented, I do not think that I can even think of having an extra marital affair, if we are having a good relationship with my husband.” (CF18) “SASA! even tries to show a woman ways of preventing HIV/AIDS in cases where there is domestic violence. A man may come home drunk or from other women and on his return wants to force his wife into sex. SASA! will already have equipped this woman will skills for safety planning. She can make an alarm, bang the door or something else that helps her to prevent herself acquiring HIV/AIDS.” (CA28M Male) “SASA! [shows] how violence leads to HIV/AIDS, they link the two. They do not treat but their medicine is to teach about prevention of violence. It is something that people never thought of in the past. The SASA! approach even took long to start, if people had know about the linkage between violence and HIV, I think HIV/AIDS would not be as much as it is today.” (CA1F Female)
Focus on prevention	“The SASA approach is preventive. Other [HIV/AIDS] organisations will wait for you until you are infected and then start helping you.” (CA6M Male) “It is difficult to wear a condom for a year. Some don't want to wear it for a night or even just one round of sex … SASA! engages the marrieds, it makes you stick to your partner peacefully because it gives you the tricks to use. It will tell you, fine you will have misunderstandings but if you have them, do this and this so instead of the man remaining in the bar seeing other women or going back home when still angry to beat his spouse he will instead say I am going back home to find a very loving woman. Those tricks from SASA! are what made the difference.” (CA7M Male)
Targets the root cause of HIV infection	“SASA! has fought against violence. In a way it reduces the rate of HIV/AIDS better than our colleagues because they only counsel but don't go deep into the cause [of infection]. Where other organisations counsel and say do this, don't do that, they do not tackle what causes a person to do those thing but SASA! goes into that.” (CA24M Male) “I am proud that this organisation really works because it has come down to the grassroots to start digging from deep in the ground, not slashing just the leaves.” (CA15M Male)
Community-based	“Other organisations go through TV and radio programs which only appeal to a few and not many people have time to listen to radios or even watch TV but the SASA! team will follow people where they are and put on the trainings closer to their homes or working places.” (CM8 Male) “SASA! has really worked because we are always with people in communities and have been in these communities for a long time. We just don't come, train and go, rather we live within these very communities.” (CA13M Male)
Trusted	“I would say that the SASA! approach differs in a way that for us we move from house to house but other organisations don't do this. Once in a while they come and gather people and talk about HIV but we do it every day, we talk to people all the time and because of this people come and approach you. It is hard for them to approach people from other organisations, they look like visitors.” (CA23F Female) “We show them that it is a community problem not a personal concern and we encourage them to test. We can even offer to accompany them to places where it will be convenient for them to test.” (CA24M)
CODES	CF (community member female) CM (Community member male) CA*F (community activist or local leader female) CA*M (community activist or local leader male)

## Discussion

This paper summarizes findings on secondary outcomes from the SASA! trial, comparing data on the quality of primary relationships and reported patterns of HIV-related risk behaviours among women and men in intervention and control communities. The findings suggest that men in intervention communities were more likely than their control counterparts to report a broad range of positive HIV-related risk behaviours and better relationship dynamics. Impacts were more limited among women, especially in relation to HIV-related risk behaviours. However, women in intervention communities felt more able to refuse sex with their partners than women in control communities, a very significant impact in settings where there is a pervasive sense of male entitlement to sex within relationships, and women have limited control over sex. They also reported more equitable relationship dynamics, especially in relation to joint decision-making and more open communication with their partners with broader impacts seen among women reporting at least moderate exposure to SASA!.

Concerning pathways through which change is occurring, some participants credit SASA! for improving the quality of their relationship, and thus increasing their willingness to test for HIV and share their results with their partners. Some also described how improved relationships manifested in improved communication, negotiation and agreement on a number of important HIV-related risk behaviours, including the use of condoms; when to have sex; and the need to be faithful to one another. However, not all relationships experienced change with important barriers preventing some participants, particularly men, from making changes that affected both HIV-related risk behaviours and relationship dynamics.

The findings also indicate that SASA! is valued, and considered more effective than other HIV prevention efforts, for its consistent presence in participants’ local communities and its delivery by known and trusted leaders. SASA!'s programming on the interconnected relationship between IPV and HIV is considered novel and thought provoking. It is also practical, offering participants advice and support on *how* to improve their relationships, and as a result, reduce HIV-related risk behaviours.

### Findings in relation to other literature

Many interventions have sought to reduce HIV-related risk behaviours or IPV in a variety of populations. Relatively few have sought to reduce HIV-related risk *and* IPV within the same population at the same time. Evidence from studies in sub-Saharan Africa including the *“*Intervention with Microfinance for AIDS and Gender Equity” and “Stepping Stones” in South Africa have demonstrated the potential for interventions to address inequitable gender norms and reduce important HIV-related risk factors including IPV [16,17]. These outcomes have however been restricted to programme participants.

To our knowledge, SASA! is the only community mobilization intervention in a low- or middle-income country that seeks to engage communities to change harmful social norms and address power imbalances between women and men that perpetuate IPV and HIV risk. As such, this study provides the only rigorous, mixed-methods evidence that evaluates the potential for community mobilization interventions to improve relationship dynamics and reduce HIV-related risk behaviours.

### Strengths and limitations

This study has a number of strengths. The randomized design prevents programme placement bias, and the measurement of outcomes among a random sample of community members along with the use of an ITT analysis mean we have been able to assess the overall community impact of the intervention rather than effects among self-selecting participants. The repeated cross-sectional design allowed us to control for baseline imbalances between intervention and control communities, and consider secular changes which occurred during the study period. Furthermore, the qualitative data provide important insights into pathways of change.

The study also has limitations. Various factors may have biased estimates of intervention effects towards the null. These include potential contamination of control sites, which, despite the presence of geographical buffers between sites, is a possibility – social diffusion is at the heart of the SASA! intervention, and the overall study area is small. Also, interruptions to programming caused by political unrest mean levels of intervention exposure might not have been optimal. Furthermore, the small number of clusters involved means the study had low power to detect statistically significant effects for many of these secondary outcomes.

Information bias is also a concern in a study of sensitive topics. While questionnaire design and interviewer training were tailored to minimize this, it is possible that such bias could have caused us to overestimate impact with respect to certain outcomes. As already discussed, for behavioural outcomes pertaining to the couple, such as condom use within the partnership, joint decision-making and male participation in traditionally female household duties, men reported higher absolute levels of positive outcomes than women. While the data collected do not come from men and women from the same partnerships, in the absence of reporting bias we might nevertheless expect the figures to be somewhat similar for men and women. With respect to the condom use outcomes, these differences may in part have arisen if men, though asked about condom use with their regular partner, reported use with extra-spousal partners. However, the difference is more likely to have arisen if men are more prone to social desirability bias than women. Greater male/female differences in intervention communities than in control communities, and the related larger effect sizes for these outcomes among men compared to women, would further suggest that this reporting bias among men could be exacerbated by exposure to the intervention. While this means observed effect sizes among men may be exaggerated, it is encouraging that results for men and women nevertheless point in the same direction – suggesting that some degree of change did occur even if estimates are somewhat inflated for men. Indeed, an increase in social desirability bias in intervention communities is an interesting result in its own right, pointing towards a shift in respondents’ perceived social norms surrounding these outcomes. This itself is an important impact, given that social norm change is a fundamental objective of the SASA! intervention, and that in the study context, reductions in many HIV-related risk behaviours (e.g. increased condom use or reduced sexual concurrency) are male-controlled behaviours. Future research should however focus more on developing indicators that are less sensitive to desirability bias. This would be particularly important for evaluation studies where the bias operates in the same direction as the expected intervention effect.

The qualitative data are also limited by the fact that the findings are restricted to people who reported change in their relationship. As such, we are unable to fully examine the barriers to change or indeed contrast the experience of couples who experienced change with those who did not to better understand facilitators and barriers to change. As with the quantitative data, the qualitative data reflect the account of only one member of a couple. This limits our ability to explore whether their partners would ascribe changes in their relationship to the same factors as the study participants. This may mean that shifts in relationship dynamics or HIV-related risks behaviours might have arisen for reasons unrelated to SASA!; for example, if men reduced concurrent partnerships because of their inability to meet gendered expectations that dictate male financial provision in relationships. Future research on pathways of change would benefit from interviewing both members in a couple in order to explore how they describe and attribute any changes in their relationship.

## Conclusions

This is the first trial to assess the community-level impact of a social change intervention focused on gender relations, violence and HIV-related risk behaviours. The findings illustrate the potential for SASA! to improve relationship dynamics and reduce HIV-related risk behaviours within intimate partnerships, with all outcomes for both men and women shifting in the hypothesized direction, and most of the reported outcomes for men being statistically significant. Even if some of this impact reflects respondent bias, given the study context, in which patriarchy is a dominant aspect, the shifts among men in intervention communities provide encouraging evidence to suggest that SASA! may be making men more cognizant of what they should or could be doing in order to foster more equitable relationships. Particularly for male-controlled indicators, changes in male behaviour have the potential to improve relationship dynamics and reduce HIV risk-related behaviours. As such programming on IPV and gender equity could play an important role in HIV prevention efforts particularly where they seek to address normative gender and relationship norms.

## Figures and Tables

**Figure A1:**
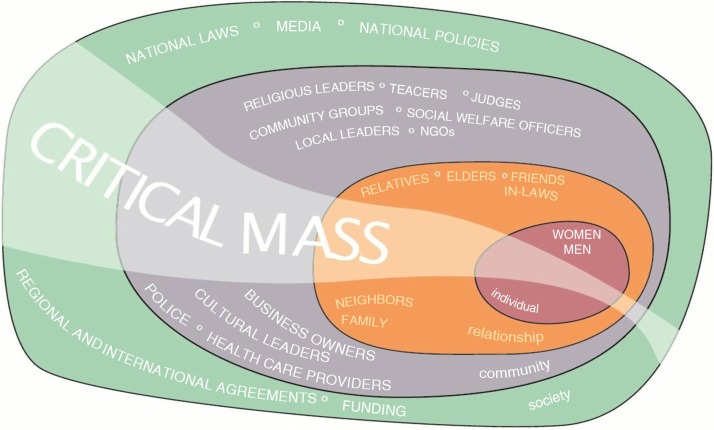
SASA! part of the fabric of the community.

## Appendices

Appendix 1. SASA! An activist kit for preventing violence against women and HIV

*Raising Voices and CEDOVIP*

Raising Voices and the Centre for Domestic Violence Prevention (CEDOVIP) are NGOs in Uganda that have been working in the field of violence prevention for over a decade. SASA! was designed by Raising Voices, an organization that strives to influence the power dynamics that shape relationships, particularly between women and men, girls and boys and adults and children. SASA! is implemented in Kampala by CEDOVIP through a team of dedicated activists who support the implementation process through daily visits to the community and building relationships with individuals and institutions in the communities. CEDOVIP is funded by a variety of donors, including Irish Aid, an anonymous donor, the United Nations Development Fund and the Elton John AIDS Foundation.

*The SASA! approach*

SASA! is a community mobilization intervention that seeks to change community attitudes, norms and behaviours that result in gender inequality, violence and an increased HIV vulnerability for women. Designed around the Ecological Model of violence [15,31], SASA! recognizes that intimate partner violence results from the complex interplay of factors which operate at the individual, relationship, community and societal levels, and that if effective change is to be achieved, it is important for interventions to systematically work with a broad range of stakeholders within the community. As such, SASA! works with every level of the community in order to build a critical mass in order to support change, as summarized in Figure A1.

*SASA! study: SASA! implementation*

In the SASA! Intervention, the CEDOVIP staff worked with four groups of actors: community activists (CAs) selected from the more progressive men and women rooted in the community; local community leaders including *Ssengas* (traditional marriage counsellors), religious, cultural and governmental leaders; professionals such as health care providers and police officers and institutional leaders who have the power to implement policy changes within their institutions.
**Community Activists**: worked voluntarily to facilitate and promote SASA! activities;
**Local community Leaders**: were encouraged to integrate a gender and power analysis into their leadership roles
**Professionals**: were trained and supported to integrate gender and power analyses in order to improve their direct prevention and response services to community members and the local community.
**Institutional leaders**: were engaged in a series of seminars introducing similar ideas and analysis of the role their sector could play in addressing violence against women

SASA! entailed the selection, training and on-going mentoring and skill building of these individuals and groups to help improve their knowledge, and inspire their activism to engage their social networks and different spheres of influence to address gender inequality and violence.

SASA! was developed from years of experience and learning from its predecessor, an intervention known as “Mobilising Communities to Prevent Violence against Women” which was piloted and run in Kawempe, an administrative division of Kampala that was not involved in the SASA! study. This experience highlighted that an explicit focus on “gender” was off-putting to many, and as such, the central focus of SASA! was to promote a critical analysis and discussion of power and power inequalities. As all community members are likely to have been disempowered at some point in their lives, this focus supported the broader engagement of both women and men in intervention activities.

In implementation, SASA! aimed to be aspirational, and support two main processes. First, it sought to inspire a critical analysis of the ways in which men and women may misuse power and how this affects their intimate relationships and the community. Second, it also supported people to explore how they can use their power positively to affect and sustain change at an individual and community level. Ultimately, power was used as an entry point in discussions about gender inequality and violence, in order for these topics to emerge from the analysis of who holds power in the community and how it may be misused, rather than being imposed on the community from the outset.

Using this operational model, SASA! supported a phased, community-level process of change, analogous to the processes set out in the individual-level behaviour-change Stages of Change Theory of Prochaska and Velicer [32]. SASA! involved four phases: Start, Awareness, Support and Action. Each of these four phases built upon the other, with an increasing number of individuals and groups involved in each phase, strengthening the critical mass committed to, and able to, create social norm change.

During the first phase (**Start**), CEDOVIP staff focused on strengthening the capacity and “power within” CAs and other key stakeholders to work on issues of violence and gender – engaging them in critical thinking and discussion about what constitutes violence; the causes and consequences of violence; the underlying links between violence, gender inequality and the misuse of power; and the implications for individuals, families and communities. Gender inequality and social norms around sexual behaviour for men and women were also discussed and opened-up to analysis. Time was also spent getting to know more about the community's perceptions of violence against women, gender and HIV, and building relationships with leaders and key gate-keepers who would support and enable community mobilisation in the subsequent phases.

During the second phase (**Awareness**) and subsequently, CAs and leaders were supported by CEDOVIP staff to conduct a range of local activism activities, including door-to-door visits, interactive community dramas, film shows, poster discussions, public events and one-on-one “quick chats.” The process of engagement for this and subsequent phases was done in an informal manner, with CAs integrating the activities into their day-to-day lives in their communities with neighbours, friends and other groups to which they belonged. The intention of this awareness phase was to spark and actively diffuse critical thinking among community members about men's use of “power over” women and the community's silence about this. The aim was for a range of different community members to engage in discussions about power, and the ways in which power imbalances between men and women help perpetuate violence against women and HIV/AIDS risk. They were also supported to question the legitimacy of violence against women and gender inequality. Alongside this, local leaders, the police, health workers and other professionals received training and support to improve their community-based prevention efforts and the provision of services. At an institutional level, leadership of police and health care sectors were engaged in a series of seminars introducing similar ideas and analysis of the role their sector could play in addressing violence against women.

In the third phase (**Support**), community members were encouraged to explore alternatives to the status quo that would create more gender equality, power balance and happiness in their families and communities. The concept of joining “power with” others was explored through the local activism activities. Here, a key theme was the power to create positive change through which community members were supported to reach out and support women experiencing violence, couples that were trying to balance power and challenge men using violence. Activities focused upon helping people to develop appropriate skills to reduce inequities in their relationships, and to challenge and respond appropriately to violence in their communities. These activities sought to encourage recognition of the ways in which different individuals can address the misuse of power, gender inequality and violence and the mutual support that can be used as a resource when communities join together with a common aim. Community leaders and professionals were also supported to work more closely together to address violence and gain skills in preventing and responding to violence against women. With support from the CEDOVIP staff, professionals, including healthcare workers and the police, examined their institutional policies and practices to identify areas where changes could be made in order to increase the capacity of the police and health sector to meaningfully respond to violence against women.

The focus of the final phase (**Action**) was to consolidate and further develop norms around non-violence and more balanced power. This sought to demonstrate the potential for more equal relationships to result in reduced violence against women and HIV/AIDS risk. The emphasis of this phase was to encourage community members, leaders, professionals and institutions to use their “power to” take action to address gender inequality and violence. Special emphasis was placed on formalizing change within community groups, local leadership structures, service delivery points and institutions.

SASA! *coverage*

As a community mobilization intervention that incorporates social diffusion, knowing the exact number of individuals that were exposed to SASA! is difficult, particularly as some individuals may have been exposed to the ideas of SASA! through their normal interactions in their communities, without having themselves directly attended any SASA! activities. That said, the findings of the on-going operational research (which incorporated over 6000 processes reports, over 750 impact monitoring reports and six rapid assessment surveys) indicate that during the period of implementation, over 400 activists (“regular” women and men in community, local government and cultural leaders, *Ssengas*, police, health care providers, drama group members) led over 11,000 activities (community conversations, door-to-door discussions, quick chats, trainings, public events, poster discussions, community meetings, film shows, drama performances) and reached more than 260,000 community members.

**Appendix 2:** Outcome measures – measured among non-polygamous respondents with a regular partner in the past year, relating to past year behaviours/experiences

Indicator	Measure	Expected direction of change due to intervention
Past year experience of sexual IPV	Reports that her partner/most recent partner has done at least one of the following things to her in the past year: Forced her to have sexual intercourse by physically threatening her, holding her down or hurting her in some way She had sexual intercourse because she was intimidated by him or afraid he would hurt her	Decrease
Feels able to refuse sex with partner	Answers “yes” to: Have you felt you could refuse to have sex with your partner/most recent partner if you do not feel like it?	Increase
Either respondent or partner initiated discussion about condom use	Answers “yes” to one or both of the following: Have you initiated a discussion about condom use with your partner/most recent partner? Has your partner/most recent partner initiated a discussion about condom use with you?	Increase
Used condom in past year	Answers “yes” to: Thinking about your partner/most recent partner, have you used a condom in the last 12 months?	Increase
Used condom at last intercourse	Answers “yes” to: Thinking about your partner/most recent partner, did you use a condom the last time you had sex?	Increase
Past year concurrent sexual partners	Answers “yes” to: Have you had a sexual relationship with any other woman/man in the last 12 months, while being with your partner/most recent partner?	Decrease
Discussed HIV testing with partner in past year	Answers “yes” to: In the last 12 months, have you talked with your partner about having an HIV test?	Increase
Had HIV test in past year	Answers “yes” to one of the following: Did one or both of you go for testing after you spoke? *(if “yes” to having talked with partner in the last 12 months about testing)* I don't need to know the results, but have you been tested for HIV in the last 12 months?	Increase
Drunk at least once a month	Answers “most days”/“weekly”/“once a month” to: In the last 12 months, how often have you been drunk? Would you say most days, weekly, once a month, less than once a month, or never?	No change
Made important decisions jointly with partner all/most of the time	Answers “all the time”/“most of the time” to: Have you made decisions jointly with your partner/most recent partner on important issues such as where you stay/live or what school the children attend? Would you say – all the time; most of the time; only sometimes; never; no important decisions in last 12 months?	Increase
Male partner helps with housework	Female respondent: Answers “yes” to: Has your partner/most recent partner regularly helped with any of the household work? Male respondent: Answers “yes” to: Have you regularly helped your partner/most recent partner with any of the household work?	Increase
Male partner helps look after children	Female respondent: Answers “yes” to: Has your partner/most recent partner regularly helped take care of the children, like feeding or bathing them? Male respondent: Answers “yes” to: Have you regularly helped your partner/most recent partner take care of the children, like feeding or bathing them?	Increase
Shown appreciation many times for work partner does in the house	Answers “many” to: How many times have you shown appreciation for the work your partner/most recent partner does inside the home? (None/a few/many/not living with partner)	Increase
Shown appreciation many times for work partner does outside the house	Answers “many” to: How many times have you shown appreciation for the work your partner/most recent partner does outside the home? (None/a few/many/not living with partner)	Increase
Discussed number of children you like to have	Answers “yes” to: Have you and your partner/most recent partner discussed how many children you would like to have/if any?	Increase
Openly asked what partner likes during sex	Answers “yes” to: Thinking back over the last 12 months, have you openly asked your partner/most recent partner about what he/she likes during sex?	Increase
Openly told partner what you like during sex	Answers “yes” to: Thinking back over the last 12 months, have you openly told your partner/most recent partner about what you like during sex?	Increase
Discussed things that happen to both you and partner during day	Answers “yes” to both: In the last 12 months, do/did you and your partner/most recent partner discuss the following topics together: Things that happen to you during the day? Things that have happened to him/her in the day?	Increase
Discussed your worries/feelings	Answers “yes” to: In the last 12 months, do/did you and your partner/most recent partner discuss the following topics together: Your worries or feelings?	Increase

## References

[1] Devries KM, Mak JYT, García-Moreno C, Petzold M, Child JC, Falder G (2013). The global prevalence of intimate partner violence against women. Science.

[2] Ellsberg M, Jansen HA, Heise L, Watts CH, Garcia-Moreno C (2008). Intimate partner violence and women's physical and mental health in the WHO multi-country study on women's health and domestic violence: an observational study. Lancet.

[3] Stöckl H, Devries K, Rotstein A, Abrahams N, Campbell J, Watts C (2013). The global prevalence of intimate partner homicide: a systematic review. Lancet.

[4] Devries KM, Mak JY, Bacchus LJ, Child JC, Falder G, Petzold M (2013). Intimate partner violence and incident depressive symptoms and suicide attempts: a systematic review of longitudinal studies. PLoS Med.

[5] Garcia-Moreno C, Jansen H, Ellsberg M, Heise L, Watts C (2006). Prevalence of intimate partner violence: findings from the WHO multi-country study on women's health and domestic violence. Lancet.

[6] Maman S, Campbell J, Sweat MD, Gielen AC (2000). The intersections of HIV and violence: directions for future research and interventions. Soc Sci Med.

[7] Jewkes RK, Dunkle K, Nduna M, Shai N (2010). Intimate partner violence, relationship power inequity, and incidence of HIV infection in young women in South Africa: a cohort study. Lancet.

[8] Dunkle KL, Jewkes RK, Nduna M, Levin J, Jama N, Khuzwayo N (2006). Perpetration of partner violence and HIV risk behaviour among young men in the rural Eastern Cape, South Africa. AIDS.

[9] Kouyoumdjian FG, Calzavara LM, Bondy SJ, O'Campo P, Serwadda D, Nalugoda F (2013). Intimate partner violence is associated with incident HIV infection in women in Uganda. AIDS.

[10] Joint United Nations Programme on HIV/AIDS (2012). Global report: UNAIDS report on the global AIDS epidemic.

[11] Maman S, Mbwambo J, Hogan N, Kilonzo G, Sweat M (2001). Women's barriers to HIV-1 testing and disclosure: challenges for HIV-1 voluntary counselling and testing. AIDS Care.

[12] World Health Organization (2006). Addressing violence against women in HIV testing and counselling: a meeting report.

[13] Dunkle KL, Jewkes RK, Brown HC, Gray GE, McIntryre JA, Harlow SD (2004). Gender-based violence, relationship power, and risk of HIV infection in women attending antenatal clinics in South Africa. Lancet.

[14] World Health Organization, London School of Hygiene and Tropical Medicine (2010). Preventing intimate partner violence against women: taking action and generating evidence.

[15] Heise L (2011). What works to prevent partner violence: an evidence overview.

[16] Pronyk P, Hargreaves J, Kim J, Morison L, Phetla G, Watts C (2006). Effect of a structural intervention for the prevention of intimate-partner violence and HIV in rural South Africa: a cluster randomised trial. Lancet.

[17] Jewkes R, Nduna M, Levin J, Jama N, Dunkle K, Puren A (2008). Impact of stepping stones on incidence of HIV and HSV-2 and sexual behaviour in rural South Africa: cluster randomised controlled trial. BMJ.

[18] Verma R, Pulerwitz J, Mahendra VS, Singh AK, Das SS, Mehra S (2008). Promoting gender equity as a strategy to reduce HIV risk and gender-based violence among young men in India.

[19] Pronyk P, Kim J, Abramsky T, Phetla G, Hargreaves J, Morison L (2008). A combined microfinance and training intervention can reduce HIV risk behaviour in young female participants. AIDS.

[20] Kim J, Watts C, Hargreaves J, Ndhlovu L, Phetla G, Morison L (2007). Understanding the impact of a microfinance-based intervention on women's empowerment and the reduction of intimate partner violence in South Africa. Am J Public Health.

[21] Michau L (2008). The SASA! Activist kit for preventing violence against women and HIV.

[22] Abramsky T, Devries KM, Kiss L, Nakuti J, Kyegombe N, Starmann E (2014). Findings from the SASA! Study: a cluster randomised controlled trial to assess the impact of a community mobilisation intervention to prevent violence against women and reduce HIV risk in Kampala, Uganda. BMC Med.

[23] Abramsky T, Devries K, Kiss L, Francisco L, Nakuti J, Musuya T (2012). A community mobilisation intervention to prevent violence against women and reduce HIV/AIDS risk in Kampala, Uganda (the SASA! Study): study protocol for a cluster randomised controlled trial. Trials.

[24] Uganda Bureau of Statistics (UBOS), Macro International Inc (2007). Uganda Demographic and Health Survey 2006.

[25] Uganda Ministry of Health, ICF International (2012). 2011 Uganda AIDS indicator survey: key findings.

[26] Garcia-Moreno C, Jansen H, Ellsberg M, Heise L, Watts C (2005). WHO multi-country study on women's health and domestic violence against women.

[27] Stata Corp (2012). Intercooled Stata.

[28] QSR International Pty Ltd (2012). NVivo qualitative data analysis software.

[29] Watts C, Heise L, Ellsberg M, Garcia-Moreno C, WHO (1999). Putting women's safety first: ethical and safety recommendations for research on domestic violence against women.

[30] Kyegombe N, Starmann E, Devries KM, Michau L, Nakuti J, Musuya T (2014). “SASA! is the medicine that treats violence.” Qualitative findings on how a community mobilisation intervention to prevent violence against women created change in Kampala, Uganda. Glob Health Action.

[31] Dahlberg LL, Krug EG, Krug EG, Dahlberg LL, Mercy JA, Zwi AB, Lozano R (2002). Violence – a global public health problem. World report on violence and health.

[32] Prochaska JO, Velicer WF (1997). The transtheoretical model of health behaviour change. Am J Health Promot.

